# Identification of aberrantly expressed long non‐coding RNAs in ovarian high‐grade serous carcinoma cells

**DOI:** 10.1002/rmb2.12330

**Published:** 2020-05-28

**Authors:** Kengo Nakashima, Shun Sato, Isao Tamura, Maki Hayashi‐Okada, Tetsuro Tamehisa, Takuya Kajimura, Kotaro Sueoka, Norihiro Sugino

**Affiliations:** ^1^ Department of Obstetrics and Gynecology Yamaguchi University Graduate School of Medicine Ube Japan

**Keywords:** long non‐coding RNAs (lncRNAs), MEG3, ovarian cancer, ovarian high‐grade serous carcinoma (HGSC), POU5F1P5

## Abstract

**Purpose:**

To identify the aberrantly expressed long non‐coding RNAs (lncRNAs) in ovarian high‐grade serous carcinoma (HGSC).

**Methods:**

Total RNA was isolated in HGSC cell lines, ovarian surface epithelial cells, and normal ovaries. Aberrantly expressed lncRNAs in HGSC were identified by PCR array, which analyzes 84 kinds of lncRNAs. To infer their functions, HGSC cell lines with different levels of expression of the identified lncRNAs were established, and then, activities of proliferation, migration, and apoptosis were examined. Expression levels of the identified lncRNAs were also examined in multiple ovarian HGSC tissues.

**Results:**

Ten aberrantly expressed lncRNAs, six upregulated and four downregulated, were identified in the HGSC cell lines. The authors established four HGSC cell lines: in two of the cell lines, one of the upregulated lncRNAs was knocked down, and in two other cell lines, one of the downregulated lncRNAs (MEG3 and POU5F1P5) was overexpressed. Migration activities were inhibited in the HGSC cell lines overexpressing MEG3 or POU5F1P5 while there were no differences in proliferation and apoptosis between the established and control cell lines. The four lncRNAs downregulated in the HGSC cell lines were also observed to be downregulated in ovarian HGSC tissues.

**Conclusion:**

The authors identified four downregulated lncRNAs in ovarian HGSC.

## 1 INTRODUCTION

Ovarian cancer is the most lethal gynecologic cancer. Based on histopathology and molecular genetics, epithelial ovarian cancers (EOCs) are mainly classified into endometrioid, clear cell, mucinous, low‐grade serous, and high‐grade serous carcinomas (HGSC).^1^, ^2^, ^3^ HGSC is the most aggressive subtype and is responsible for approximately two‐thirds of total EOCs.^2^ These subtypes of EOCs have different histological features and can be considered as inherently different diseases, depending on their origin and genetic background.^2^, ^3^ Despite recent development in surgery and chemotherapy, the 5‐year survival rate of ovarian cancer is still only 30%‐40% owing to late diagnosis and chemoresistance or relapse.^4^ Therefore, to improve prognosis, it is necessary to elucidate the molecular mechanisms involved in the progression and malignant behaviors of ovarian cancers.

In recent years, with the advances in large‐scale sequencing technology, it has been widely accepted that more than 98% of the human genome is transcribed into non‐coding RNAs that do not encode obvious proteins.^5^, ^6^, ^7^ A type of non‐coding RNA, long non‐coding RNA (lncRNA), consists of transcripts longer than 200 nucleotides in length. lncRNAs have roles in a variety of biological processes such as X chromosome inactivation, genomic imprinting, chromatin remodeling, and cell fate determination.^8^, ^9^ In addition, lncRNAs are closely associated with tumorigenesis in various types of cancers. In fact, the number of publications related to the biological roles of lncRNAs in cancers has increased exponentially in the past few years.^7^, ^10^ In ovarian cancer, the lncRNAs such as H19, HOTAIR, HOXA11‐AS, LSINCT5, MALAT1, and PVT1 have been reported to be involved in cell proliferation and malignancy.^10^, ^11^, ^12^, ^13^

In this study, we focused on HGSCs, which are the most frequently occurring and aggressive subtypes among ovarian cancers, and aimed to identify lncRNAs involved in the progression and malignant behaviors of HGSC. We compared lncRNA expression profiles between HGSC cell lines and normal ovarian cells and ovarian tissue samples, and identified the aberrantly expressed lncRNAs specific to the HGSC cell lines. In addition, some of the lncRNAs downregulated in the HGSC cell lines were observed to be downregulated in ovarian HGSC tissues. Our results also suggest that some of the identified lncRNAs have roles in malignant behaviors.

## 2 METHODS

### 2.1 Cell culture and tissues samples

Human ovarian cancer cell lines, KURAMOCHI and TYK‐nu, were used as HGSC cell models. Both cell lines were purchased from Japanese Collection of Research Bioresources Cell Bank. KURAMOCHI and TYK‐nu cells were cultured in RPMI‐1600 (Wako) and eagle’s minimal essential medium (Sigma‐Aldrich) supplemented with 10% fetal bovine serum, respectively. Human ovarian surface epithelial cells (HOSE) were purchased from Cosmo‐bio and cultured in ovarian surface epithelial cell medium (ScienCell Research Laboratories).

Twenty‐two cases of HGSC specimens were obtained from the patients who underwent operation at Shimane University hospital. Ten cases of normal ovarian tissues were obtained from the patients who underwent oophorectomy by a reason different from HGSC at Yamaguchi University hospital. Dissected specimens were immediately immersed in liquid nitrogen and stored at −80°C until RNA isolation.

### 2.2 RNA isolation and real‐time reverse transcription polymerase chain reaction (RT‐PCR)

Total RNA was isolated from the cell lines using an RNeasy mini kit (Qiagen) according to the manufacturers’ instructions. Total RNA was also isolated from the tissue specimens by ISOGEN reagent (Nippon Gene), followed by chloroform extraction and 2‐propanol precipitation.^14^

First‐strand cDNA was synthesized from 1 μg of total RNA by random hexamers using a QuantiTect Reverse Transcription Kit (Qiagen) as previously reported.^14^ Real‐time RT‐PCR was carried out using TB green premix Ex taq II (Takara) and primer sets listed in Table 1 under the cycling condition (40 cycles of 95°C for 5 seconds and 60°C for 20 seconds with an initial step of 95°C for 10 seconds). The relative expression levels were calculated with the delta‐delta Ct method using *GAPDH* as a reference gene.

**Table 1:** PCR primers used in this study

Primer name	Usage	Forward	Reverse
CDKN2B‐AS1	RT‐PCR	ATTCCTCAGCTCCTCTCATCTG	CCAAGACAGCAAGTGGTATTGA
DLEU2	RT‐PCR	CTGGAGAACAGCCTCACTTCTT	GTAGAGGTCTCTTTTATTGTGGTCTT
LINC00152	RT‐PCR	ATGGCTTGAACATTTGGTCTTC	TTCGATCAAGTGTGTCATAGAGC
LINC01234	RT‐PCR	TGGGAAAGAGGAGTCTCTCG	ATCTGAGGAGCTTGGAATGC
ADAMTS9‐AS2	RT‐PCR	TATTGAAACCTGCTTTGTGCTG	TCATACTTTGGCATGACTGTCC
MEG3	RT‐PCR	GCCATCACCTGGATGCCTAC	AGTCTCTGGGTCCAGCCTGT
POU5F1P5	RT‐PCR	ACTGCAGCAGATCAGTCACATT	CAAAATCCTCTCATGGTGCATA
XIST	RT‐PCR	ACGCTGCATGTGTCCTTAGTAG	TTGGSGCCTCTTATAGCTGTTTG
GAPDH	RT‐PCR	AGGTGAAGGTCGGAGTCA	GGTCATTGATGGCAACAA
MEG3 vector	Construction	ATTAAGGATCCTCGAAGAGAGGGAGCGCGCCTTGG	CCTGCGGTCGCGGCCGCACACATTTATTGAGAGCACA
POU5F1P5 vector	Construction	ATTAAGGATCCTCGAATCCAGTCCCAGGACATCTC	CCTGCGGTCGCGGCCGCTCTACCTACTGTGTCCCAGT

### 2.3 PCR Array

cDNA synthesis was performed using the RT^2^ First Strand Kit (Qiagen) according to the manufacturers’ instructions.^15^, ^16^ In this study, PCR array covering 84 kinds of lncRNAs was used, which are reported to be related with variable cancers (RT^2^ lncRNA PCR Array Human Cancer Pathway Finder (Qiagen)). Real‐time PCR was carried out with the RT^2^ SYBR Green Master Mix (Qiagen) under the cycling conditions (40 cycles of 95°C for 15 seconds, 60°C for 60 seconds, with an initial step of 95°C for 10 minutes). The relative expression levels were calculated with the delta‐delta Ct method using *ACTB* as a reference gene. Significant changes in the lncRNA expression were defined as at least twofold upregulation or downregulation of ones in both two HGSC cell lines compared to those in HOSE and normal ovaries.

### 2.4 Knockdown and overexpression of the lncRNAs in a HGSC cell line (TYK‐nu)

For knockdown of LINC00152 and LINC01234, siRNAs for each lncRNA (Lincode Human lncRNAs siRNA SMART pool) and non‐targeting control (Lincode Non‐Targeting Pool siRNA) were purchased from Dharmacon. TYK‐nu cells were plated at approximately 5 × 10^4^ cells in 6‐well plates and, at 50% confluence, were transfected with the 20 nmol/L siRNAs by RNAi MAX (Invitrogen).^17^

For overexpression of MEG3 and POU5F1P5, firstly the expression vectors of each lncRNA were constructed. The full length of MEG3 and POU5F1P5 cDNA was amplified by RT‐PCR using a HOSE cDNA as a template, primers shown in Table 1, and PrimeSTAR GXL DNA polymerase (Takara), under the cycling conditions (35 cycles of 98°C for 10 seconds, 60°C for 15 seconds, and 68°C for 2.5 minutes). The amplified lncRNA fragments were inserted into multicloning site of pMXs‐IRES‐Bsd vector (Cell Biolabs) by In‐Fusion HD Cloning Kit (Takara) according to the manufacturers’ instructions.^17^ Then, the constructed MEG3 and POU5F1P5 expression vector and control vector (non‐treated pMXs‐IRES‐Bsd vector), respectively, together with the vectors expressing the retroviral constitutive proteins were co‐transfected into HEK293T cells (Takara) using the Lipofectamine 3000 (Invitrogen). Two days after the transfection, the culture medium was concentrated to 100 times and used as a packaged retrovirus. The packaged retrovirus was added to TYK‐nu cells plated at approximately 5 × 10^4^ cells in 6‐well plates. The stable cell lines were established by sorting with 2 μg/mL blasticidin S for a month.^14^, ^17^

### 2.5 Cell proliferation assay

TYK‐nu lines, in which the lncRNA expression was altered, and the control lines were plated at approximately 6 × 10^4^ cells in 6‐well plates, respectively.^18^, ^19^ At every 24 hours, single cell suspension was prepared in each line by trypsinization and was counted with TC20 Automated Cell Counter (Bio‐Rad Laboratories). Each experiment was carried out in triplicate.

### 2.6 Cell cycle assay and apoptosis assay

For cell cycle assay, TYK‐nu lines overexpressing MEG3 and POU5F1P5 and the control lines were trypsinized, fixed for 30 minutes in cold 70% ethanol, and then adjusted to a concentration of 1 × 10^6^ cells/mL in 0.25 mg/mL RNase in PBS. Resultant single cell suspension was stained with 7‐aminoactinomycin D (7‐AAD) (Bio‐Rad Laboratories) and assayed using NovoCyte Flow Cytometer (ACEA Biosciences). The percentage of cells in each cell cycle stage (G0/G1, S, G2/M phase and sub G1 population) was evaluated with a NovoExpress software (ACEA Biosciences).

For apoptosis assay, an Annexin V‐FITC Apoptosis Detection Kit (Affymetrix) was used according to the manufacturer's protocol. In brief, the cells of TYK‐nu lines overexpressing MEG3 or POU5F1P5 and the control lines were trypsinized into single cells and were stained with annexin V‐FITC and 7‐AAD. Ten thousand cells were counted, and annexin V‐positive cells were measured by NovoCyte Flow Cytometer. Cells that were annexin V‐positive and 7‐AAD‐negative, and double‐positive, were considered to be early and late apoptotic cells, respectively.

### 2.7 Wound healing assay

Cells of TYK‐nu lines overexpressing MEG3 or POU5F1P5 and the control lines were plated at approximately 2 × 10^5^ cells in 6‐well plates and cultured until they reached confluence. Linear scratch wounds were created on the cell layer in the center of each well with a 1000‐µL sterile pipette tip. After 72 hours, images were taken to evaluate the wounds at the same fields under the microscope, and the separation distances between wound sides were quantified as previously reported.^18^ Each experiment was performed in triplicate, and the mean distances were obtained from three independent experiments.

### 2.8 Migration assay

Cell migration assay was performed with a BioCoat Matrigel Invasion Chamber (Corning Life Science) according to the manufacturer's protocol as previously reported.^18^ In brief, the cells of TYK‐nu lines overexpressing MEG3 or POU5F1P5 and the control lines were trypsinized into single cells and plated at 5 × 10^4^ cells with serum‐free medium in insert chambers on the well (12‐well Transwell system) (Corning Life Science). As a chemo‐attractant, the medium containing 10% FBS was filled under the insert chamber. After 24‐hours culture, the cells on the upper surface of the insert chambers were completely removed by wiping with cotton swabs. The membranes of the insert chambers were fixed, and the migrated cells were stained with Diff‐Quick (Sysmex). The stained cells were counted at 200× magnification in five randomized field views, and the mean was calculated. Three independent experiments in triplicate were performed.

### 2.9 Statistical analysis

The significance of the difference between the two groups was analyzed by Student's *t* test and Wilcoxon test. A probability value of *P* < .05 was considered to be significant. All the statistical analyses were performed by using the SPSS 5.0 J for Windows software package (SAS Institute).

## 3 RESULTS

### 3.1 Identification of aberrantly expressed lncRNAs in HGSC cell lines

**Figure 1:**
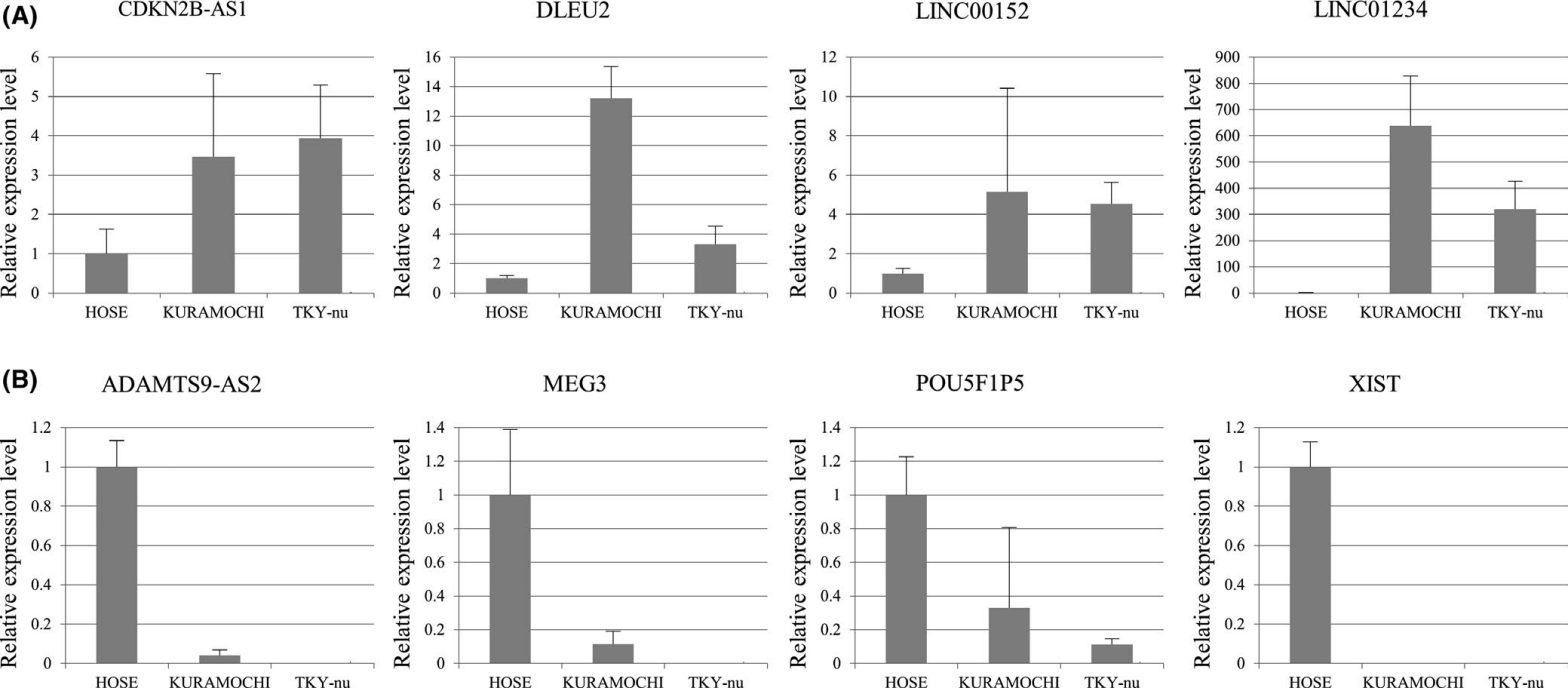
Validation of the aberrantly expressed lncRNAs in ovarian high‐grade serous carcinoma (HGSC) cell lines (KURAMOCHI and TYK‐nu) and human ovarian surface epithelial cells (HOSE). A, Expression levels of the four lncRNAs upregulated in HGSC cell lines (CDKN2B‐AS1, DLEU2, LINC00152, and LINC01234), which was analyzed by real‐time RT‐PCR in triplicates in two HGSC cell lines and HOSE. GAPDH was used as an internal control. Values are mean ± SD. B, Expression levels of the four lncRNAs downregulated in HGSC cell lines (ADAMTS9‐AS2, MEG3, POU5F1P5, and XIST), which was analyzed by real‐time RT‐PCR in triplicates in two HGSC cell lines and HOSE. GAPDH was used as an internal control. Values are mean ± SD

To identify aberrantly expressed lncRNAs in HGSC, we used HGSC cell lines because using cell lines generally yields more uniform data than using tissue specimens that reflect individual differences. To analyze lncRNAs, we used a PCR array that can comprehensively analyze the expression of 84 kinds of lncRNAs associated with various cancer types. The expression levels of the lncRNAs in the two HGSC cell lines, KURAMOCHI and TYK‐nu, were compared to those in human ovarian surface epithelial cells (HOSE) and normal ovarian tissues. Of the 84 lncRNAs analyzed, six upregulated lncRNAs (CDKN2B‐AS1, DLEU2, LINC00152, LINC01234, PRNCR1, and SNHG16) and four downregulated lncRNAs (ADAMTS9‐AS2, MEG3, POU5F1P5UCA1, and XIST) were identified as aberrantly expressed lncRNAs in the HGSC cell lines (Table 2). Next, the expression levels of the identified lncRNAs were examined by real‐time RT‐PCR in the two HGSC cell lines and HOSE. The expression profiles were verified for the eight lncRNAs except PRNCR1 and SNHG16, because PCR was not amplified by our designed primers for PRNCR1 and SNHG16 (Figure 1).

**Table 2:** Aberrantly expressed lncRNAs in ovarian high‐grade serous carcinoma (HGSC) cell lines

Symbol	Fold change comparing to HOSE		Fold change comparing to ovaries	
	KURAMOCHI	TYK‐nu	KURAMOCHI	TYK‐nu
Upregulated in HGSC cell lines				
CDKN2B‐AS1	81.99	8.52	81.99	8.52
DLEU2	65.46	7.27	18.18	2.02
LINC00152	7.58	3.34	498.86	219.51
LINC01234	57.33	51.79	187.10	169.01
PRNCR1	3.94	4.10	11.20	11.66
SNHG16	2.41	2.55	3.03	3.20
Downregulated in HGSC cell lines				
ADAMTS9‐AS2	0.01	0.02	0.00	0.00
MEG3	0.01	0.02	0.00	0.00
POU5F1P5	0.02	0.02	0.03	0.04
XIST	0.00	0.00	0.00	0.00

Note

KURAMOCHI and TYK‐nu: HGSC cell lines.

Abbreviation: HOSE, human ovarian surface epithelial cells.

### 3.2 Establishment of the HGSC cell lines in which expressions of upregulated or downregulated lncRNAs are altered

**Figure 2:**
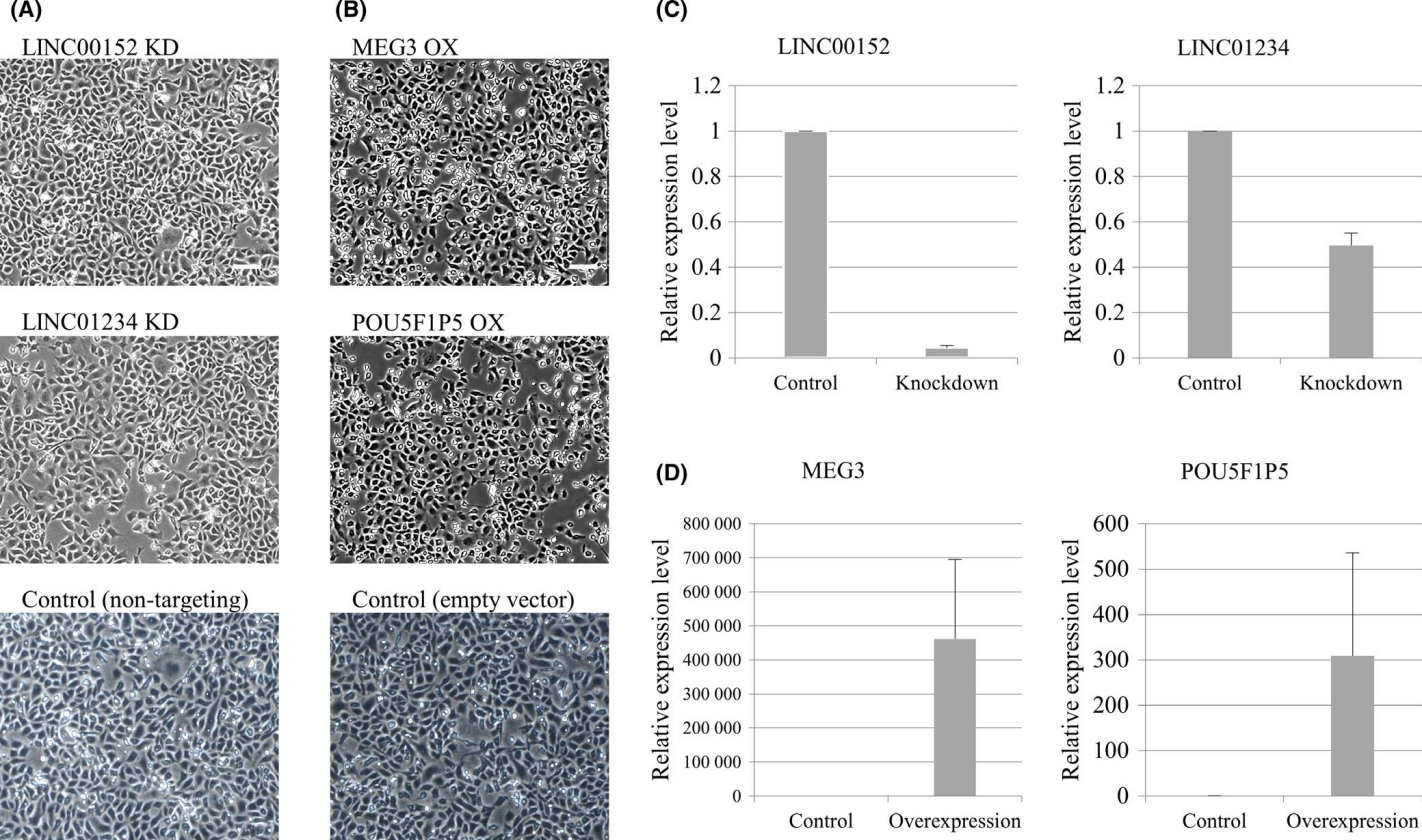
Establishment of HGSC cell lines (TKY‐nu) in which the expression of upregulated lncRNAs (LINC00152 and LINC01234) or downregulated lncRNAs (MEG3 and POU5F1P5) was altered. A, Cell morphology of the TKY‐nu line in which LINC00152 or LINC01234 was knocked down (KD). Cells transfected with non‐targeting siRNA were used as a mock control cell line (non‐targeting). Scale bars: 100 μm. B, Cell morphology of the TKY‐nu line in which MEG3 or POU5F1P5 was overexpressed (OX). Cells transduced with empty vector were used as a mock control line (empty vector). Scale bars: 100 μm. C, Confirmation of the knockdown of LINC00152 or LINC01234. Relative expression levels of LINC00152 and LINC01234 to GAPDH in each KD and control cell line were analyzed in triplicates by real‐time RT‐PCR. Values are mean ± SD. (D) Confirmation of the overexpression of MEG3 or POU5F1P5. Relative expression levels of MEG3 and POU5F1P5 to GAPDH in each overexpressing and control cell line were analyzed in triplicates by real‐time RT‐PCR. Values are mean ± SD

To investigate the function of the aberrantly expressed lncRNAs in the HGSC cell lines, we established the HGSC cell lines in which the upregulated lncRNAs are knocked down and in which the downregulated lncRNAs are overexpressed. The knockdown and overexpression succeeded in TYK‐nu, but not in KURAMOCHI because of its slow growth and low efficiency of gene transfer. Among the upregulated lncRNAs, LINC00152 and LINC01234 were knocked down by siRNA (Figure 2A) while CDKN2B‐AS1 and DLEU2 were not. The HGSC cell lines that stably overexpressed each of the downregulated lncRNAs (MEG3 and POU5F1P5) were established by transducing their expression vectors into the cells (Figure 2B). For the remaining downregulated lncRNAs, ADAMTS9‐AS2 and XIST, their expression vectors could not be constructed because their full‐length cDNA is too long to be amplified by our designed primers. Knockdown and overexpression in each of the four established cell lines were confirmed by real‐time RT‐PCR (Figure 2C and D). There were no differences in cell morphology between the established cell lines and control cell lines (Figure 2A and B).

### 3.3 Cell proliferation of the established cell lines

**Figure 3:**
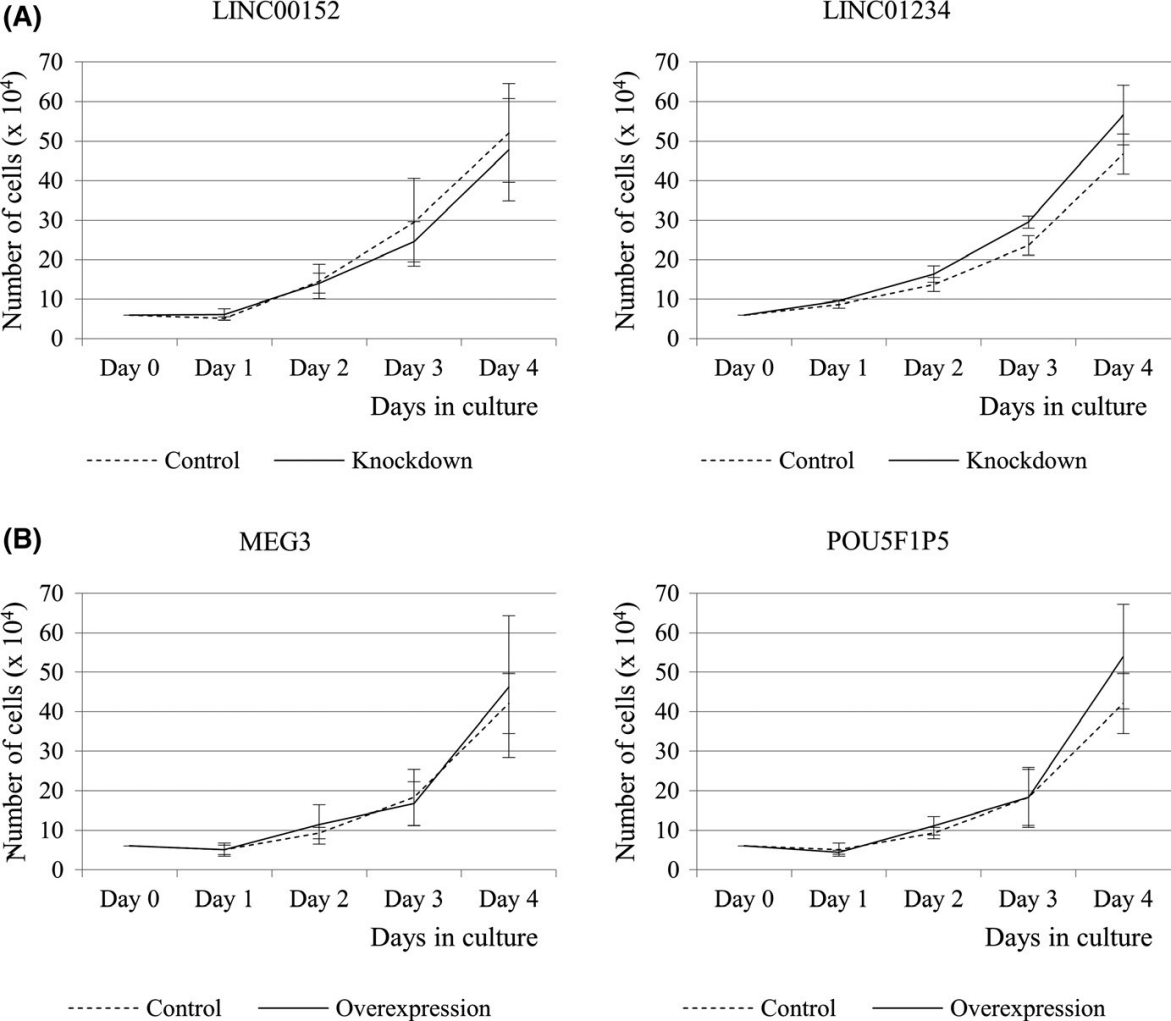
Cell proliferation assays of the HGSC cell lines established in this study. A, The cell number in each knockdown cell line (LINC00152 and LINC01234) and control cell line (non‐targeting) was counted every 24 h. Values show the mean ± SD of three independent experiments. B, The cell number in each overexpressed cell line (MEG3 and POU5F1P5) and control cell line (empty vector) was counted every 24 h. Values show the mean ± SD of three independent experiments

Cell proliferation was analyzed in the HGSC cell lines in which LINC00152 or LINC01234 was knocked down (Figure 3A) and in which MEG3 or POU5F1P5 was overexpressed (Figure 3B). There was no significant difference in cell proliferation between the established cell lines and control cell lines (Figure 3).

### 3.4 Cell cycles and apoptosis of the established cell lines

**Figure 4:**
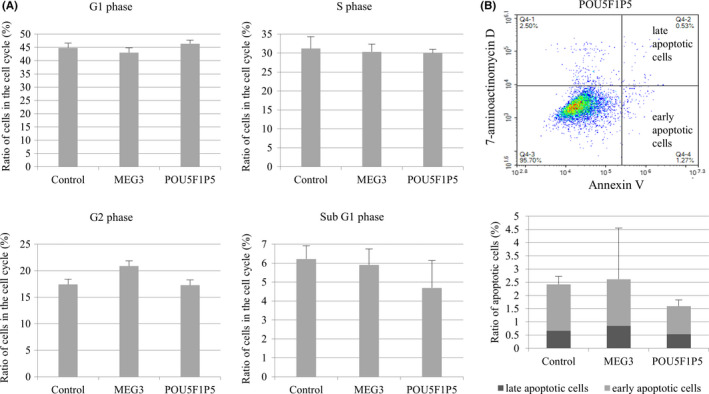
Cell cycle and apoptosis in the HGSC cell lines overexpressing MEG3 and POU5F1P5. A, Cell cycle assay in the MEG3‐ and POU5F1P5‐overexpressing and control cell lines using flow cytometer. The percentage of the cells in each cell cycle stage (G0/G1, S, G2, and sub G1 phase) were analyzed in triplicate. Values are mean ± SD. B, Apoptosis assay in MEG3‐ and POU5F1P5‐overexpressing and control cell lines using flow cytometer. The upper panel shows the flow cytometry in POU5F1P5‐overexpressing cell line as a representative result. Cells that were annexin V‐positive and 7‐aminoactinomycin D‐negative, and double‐positive, were divided into the early and late apoptotic cells, respectively. The lower panel shows the percentage of the late (black) and early (gray) apoptotic cells, which were analyzed in triplicate. Values are mean ± SD

The cell cycle and apoptosis were analyzed in the HGSC cell lines in which MEG3 or POU5F1P5 was overexpressed (Figure 4), because these cell lines are stable cell lines and a sufficient number of cells could be obtained for analysis. In the cell cycle analysis, the percentage of the cells included in each cell cycle stage (G1, S, G2, and sub G1 phase) was not significantly different between the MEG3‐ or POU5F1P5‐overexpressing cell line and the control cell line (Figure 4A). A sufficient number of the cells were not obtained to examine cell cycles and apoptosis in the HGSC cell lines in which LINC00152 or LINC01234 was knocked down because knockdown of LINC00152 or LINC01234 was transiently done by siRNA.

In the apoptosis analysis, annexin V‐positive and 7‐AAD‐negative early apoptotic cells and double‐positive late apoptotic cells were detected, respectively (Figure 4B, upper panel). The percentage of the cells included in the early and late apoptotic cell areas was not significantly different between the MEG3‐ or POU5F1P5‐overexpressing cell line and the control cell line (Figure 4B, lower panel).

### 3.5 Cell migration activities of the established cell lines

**Figure 5:**
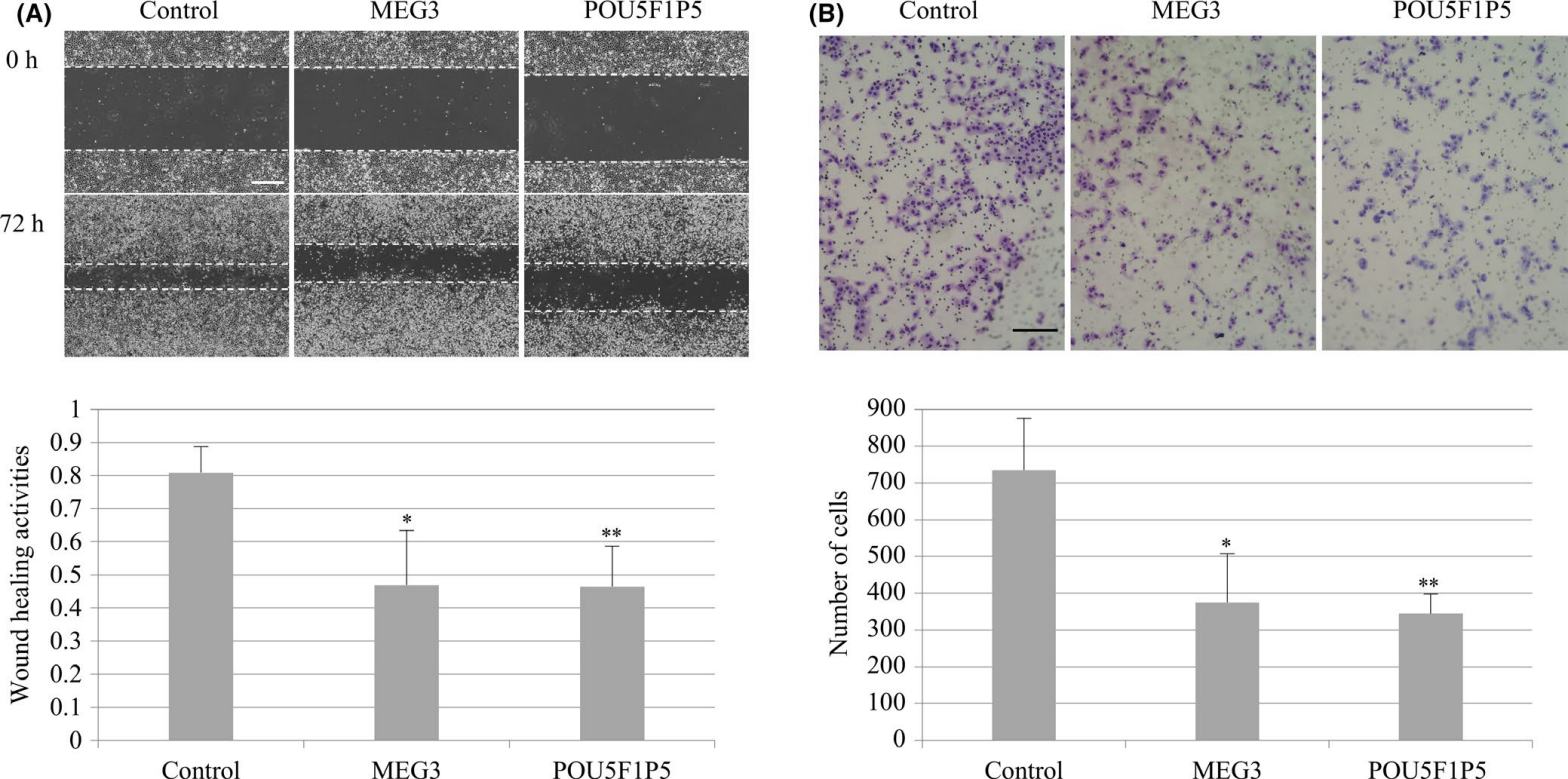
Effects of overexpression of lncRNAs (MEG3 or POU5F1P5) on the cell migration activity in the HGSC cell line. A, Wound healing assay in the MEG3‐ and POU5F1P5‐overexpressing and control cell lines. Representative photographs at the same fields, 0 and 72 h after wound creation, were shown (upper panel). Horizontal dotted lines indicate the wound margins. Scale bar: 200 µm. Wound healing activities were calculated by dividing the migration distance by the original width (lower panel). Values are mean ± SD of three independent experiments. *; *P* < .05 and **; *P* < .01 compared to the control. B, Cell migration assay in the MEG3‐ and POU5F1P5‐overexpressing and control cell lines. Representative photographs of the migrated cells stained by Diff‐Quick stain were shown (upper panel). Scale bar: 200 µm. Migration activities were calculated by counting the number of the migrated cells in five randomized fields (lower panel). Values are mean ± SD of three independent experiments. *; *P* < .05 and **; *P* < .01 compared to the control (*t* test)

Cell migration activity was assessed in the HGSC cell lines in which MEG3 or POU5F1P5 was overexpressed by two methods: a wound healing assay (Figure 5A) and a cell migration assay (Figure 5B). In the wound healing assay, in the MEG3‐ and POU5F1P5‐overexpressing cell lines, the number of cells that migrated to the wounded area was clearly lower 72 hours after wound creation (Figure 5A, upper panel), and the wound healing activity measured was significantly lower in the MEG3‐ and POU5F1P5‐overexpressing cell lines than in the control cell line (Figure 5A, lower panel). In the cell migration assay, the number of migrated cells permeating the membrane of the insert chamber was significantly lower in the MEG3‐ and POU5F1P5‐overexpressing cell lines than in the control cell line (Figure 5B). Therefore, the overexpression of these two lncRNAs inhibited the cell migratory activities in the HGSC cell lines.

### 3.6 Expression levels of the aberrantly expressed lncRNAs in ovarian HGSC tissues

**Figure 6:**
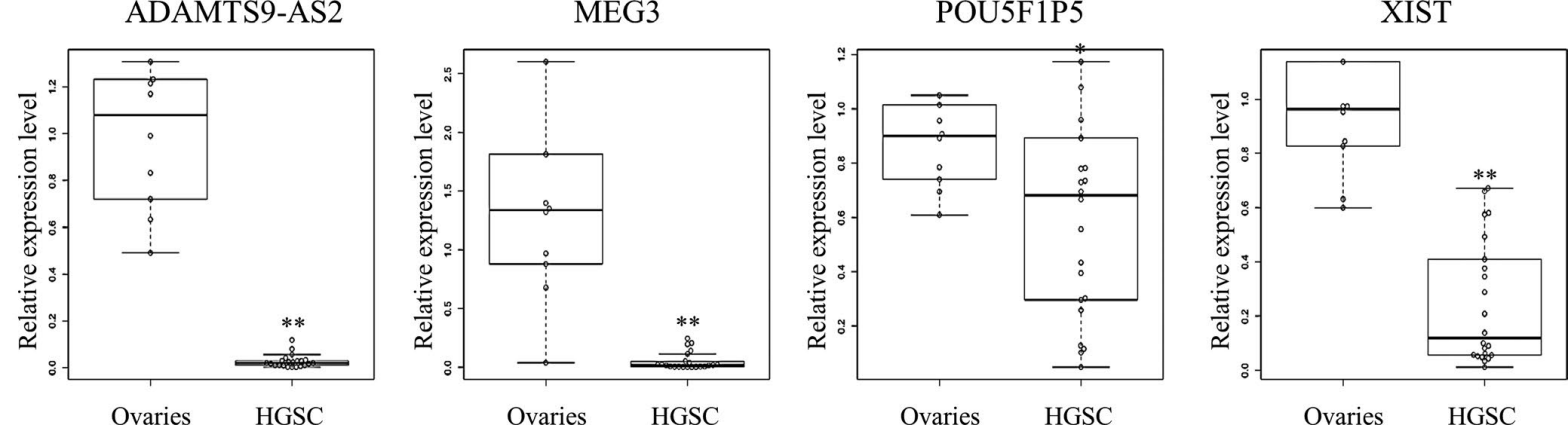
Expression levels of aberrantly expressed lncRNAs in HGSC cell lines in multiple specimens of HGSC and ovarian tissues. Box plots of relative expression levels of lncRNAs downregulated in HGSC cell lines (ADAMTS9‐AS2, MEG3, POU5F1P5, and XIST), which were analyzed by real‐time RT‐PCR in triplicates, in the twenty‐two HGSC tissues and ten ovaries. GAPDH was used as an internal control. A plotted circle indicates each sample. *P < .05 and **P < .01 compared to the control (Mann‐Whitney *U* test)

To investigate the expression profiles in ovarian HGSC tissues for the aberrantly expressed lncRNAs in the HGSC cell lines, the expression levels of four upregulated lncRNAs (CDKN2B‐AS1, DLEU2, LINC00152, and LINC01234) and four downregulated lncRNAs (ADAMTS9‐AS2, MEG3, POU5F1P5, and XIST) were examined in 22 specimens of ovarian HGSC tissues and 10 specimens of normal ovarian tissues by real‐time RT‐PCR. The expression levels of the four downregulated lncRNAs (ADAMTS9‐AS2, MEG3, POU5F1P5, and XIST) were significantly lower in ovarian HGSC tissues compared to normal ovarian tissues, confirming that the expression profiles of these four lncRNAs were consistent between the HGSC cell lines and ovarian HGSC tissues (Figure 6). However, there was no significant difference in the expression levels of four upregulated lncRNAs (CDKN2B‐AS1, DLEU2, LINC00152, and LINC01234) between ovarian HGSC tissues and normal ovarian tissues (data not shown).

## 4 DISCUSSION

In this study, based on a comparison of the expression profiles of HGSC cell lines with human ovarian surface epithelial cells (HOSE) and normal ovarian tissues, we identified ten aberrantly expressed lncRNAs in the HGSC cell lines and showed that among them four aberrantly downregulated lncRNAs (ADAMTS9‐AS2, MEG3, POU5F1P5, and XIST) were similarly downregulated in ovarian HGSC tissues. The expression levels of ADAMTS9‐AS2, MEG3, and XIST were especially low in ovarian HGSC tissues compared with normal ovaries. Therefore, they are possible candidates of HGSC‐specific lncRNAs for molecular targeting therapy in future directions. These lncRNAs offer therapeutic applications by restoration of the lncRNAs that suppress malignant behaviors in a highly cancer‐specific manner like a drug delivery system.

Some of the HGSC‐specific lncRNAs may have roles in malignant behaviors. Among four aberrantly downregulated lncRNAs (ADAMTS9‐AS2, MEG3, POU5F1P5, and XIST), MEG3 and POU5F1P5 inhibited the cell migration when they were overexpressed. This is the first report of an inhibitory effect of MEG3 and POU5F1P5 on cell migration in ovarian HGSC. However, it is unclear why MEG3 and POU5F1P5 did not inhibit cell proliferation.

POU5F1P5 has been reported to be upregulated in various cancer tissues and cancer cell lines,^20^ but the function of POU5F1P5 remains unclear. POU5F1P5 was reported to be upregulated in endometrial cancer specimens, and overexpression of POU5F1P5 promoted the proliferation of endometrial cancer cells.^21^ This is inconsistent with our result that upregulation of POU5F1P5 had tumor suppressor effects in HGSC cell lines. The discrepancy may reflect the difference in cancer types, which, if true, suggests that the function of POU5F1P5 depends on cancer type. In fact, some lncRNAs including XIST have been reported to show either suppress or promote cancer depending on the type of cancer.^7^, ^13^, ^22^, ^23^

MEG3 inhibits malignant behaviors in a variety of cancers.^10^, ^11^, ^12^, ^13^ In addition, MEG3 is downregulated in ovarian cancer tissues, and overexpression of MEG3 inhibits cell proliferation, migration, and invasion and promotes apoptosis in ovarian cancer cell lines.^24^, ^25^, ^26^ However, our results show that overexpression of MEG3 inhibited cell migration in HGSC cell lines. The discrepancy may be due to the difference in the cell lines used for functional analysis. The ovarian cancer cell lines used in the above studies are OVCAR3, SKOV3, and A2780, and they have different properties from our HGSC cell lines.^24^, ^27^ Cell lines such as SKOV3, A2780, and OVCAR3 have often been used as HGSC models in studies of ovarian cancers. Large numbers of HGSC specimens and ovarian cancer cell lines are catalogued in The Cancer Genome Atlas (TCGA) and Broad‐Novartis Cancer Cell Line Encyclopedia (CCLE) databases, respectively. A recent comparison of the data in these databases revealed that the abovementioned cell lines are not suitable as HGSC models due to differences in mutation, chromosomal copy number, and mRNA expression profiles.^27^ Therefore, we used KURAMOCHI and TYK‐nu as HGSC cell lines because their mutations and mRNA expression profiles are more similar to those of HGSC specimens.^27^

Downregulation of ADAMTS9‐AS2 or XIST is associated with progression of cell proliferation and malignant behaviors in ovarian cancer.^13^, ^22^, ^28^ Although the functions of ADAMTS9‐AS2 and XIST were not examined in this study, the remarkably low expression in the HGSC tissues suggests that ADAMTS9‐AS2 or XIST has inhibitory effects on malignant behaviors of ovarian HGSC.

About the four upregulated lncRNAs (CDKN2B‐AS1, DLEU2, LINC00152, and LINC01234), knockdown using siRNAs did not show any inhibitory effects and their expression levels were not different between ovarian HGSC tissues and normal ovarian tissues. We cannot clearly explain the difference in actions and expressions between upregulated and downregulated lncRNAs. We speculate as follows; the lncRNAs in this study were originally identified in HGSC cell lines. Although the HGSC cell lines used in this study are derived from ovarian HGSC cells, ovarian HGSC tissues reflect individual differences, indicating the heterogeneity. Therefore, the character of the HGSC cell lines is not always consistent with the character of the ovarian HGSC tissues.

In conclusion, we identified four new lncRNAs that are downregulated in ovarian HGSC. These four lncRNAs may have uses in the diagnosis and treatment of HGSC.

## CONFLICT OF INTEREST

The authors declare that there are no conflicts of interest.

## HUMAN RIGHTS STATEMENT AND INFORMED CONSENT

All of the experiments handling human tissues were conducted in accordance with the Declaration of Helsinki, and the protocol was approved by the Institutional Review Board of Yamaguchi University Graduate School of Medicine. Informed consent was obtained from the patients before the collection of any samples.

## References

[1] Seidman JD , Horkayne‐Szakaly I , Haiba M , Boice CR , Kurman RJ , Ronnett BM . The histologic type and stage distribution of ovarian carcinomas of surface epithelial origin. Int J Gynecol Pathol. 2004;23:41‐44. 14668549 10.1097/01.pgp.0000101080.35393.16

[2] Vaughan S , Coward JI , Bast RC Jr , et al. Rethinking ovarian cancer: recommendations for improving outcomes. Nat Rev Cancer. 2011;11:719‐725. 21941283 10.1038/nrc3144 PMC3380637

[3] Berns EM , Bowtell DD . The changing view of high‐grade serous ovarian cancer. Cancer Res. 2012;72:2701‐2704. 22593197 10.1158/0008-5472.CAN-11-3911

[4] Yeung TL , Leung CS , Yip KP , Au Yeung CL , Wong ST , Mok SC . Cellular and molecular processes in ovarian cancer metastasis. A review in the theme: cell and molecular processes in cancer metastasis. Am J Physiol Cell Physiol. 2015;309:C444‐456. 26224579 10.1152/ajpcell.00188.2015 PMC4593771

[5] Lorenzen JM , Thum T . Long noncoding RNAs in kidney and cardiovascular diseases. Nat Rev Nephrol. 2016;12:360‐373. 27140855 10.1038/nrneph.2016.51

[6] Djebali S , Davis CA , Merkel A , et al. Landscape of transcription in human cells. Nature. 2012;489:101‐108. 22955620 10.1038/nature11233 PMC3684276

[7] Huarte M . The emerging role of lncRNAs in cancer. Nat Med. 2015;21(11):1253‐1261. 26540387 10.1038/nm.3981

[8] Hung T , Chang HY . Long noncoding RNA in genome regulation: prospects and mechanisms. RNA Biol. 2010;7:582‐585. 20930520 10.4161/rna.7.5.13216 PMC3073254

[9] Flynn RA , Chang HY . Long noncoding RNAs in cell‐fate programming and reprogramming. Cell Stem Cell. 2014;14:752‐761. 24905165 10.1016/j.stem.2014.05.014 PMC4120821

[10] Tripathi MK , Doxtater K , Keramatnia F , et al. Role of lncRNAs in ovarian cancer: defining new biomarkers for therapeutic purposes. Drug Discov Today. 2018;23:1635‐1643. 29698834 10.1016/j.drudis.2018.04.010 PMC6139057

[11] Wang JY , Lu AQ , Chen LJ . LncRNAs in ovarian cancer. Clin Chim Acta. 2019;490:17‐27. 30553863 10.1016/j.cca.2018.12.013

[12] Zhan L , Li J , Wei B . Long non‐coding RNAs in ovarian cancer. J Exp Clin Cancer Res. 2018;37:120. 29921308 10.1186/s13046-018-0793-4 PMC6008930

[13] Meryet‐Figuière M , Lambert B , Gauduchon P , et al. An overview of long non‐coding RNAs in ovarian cancers. Oncotarget. 2016;7:44719‐44734. 26992233 10.18632/oncotarget.8089 PMC5190131

[14] Sato S , Maekawa R , Tamura I , et al. SATB2 and NGR1: potential upstream regulatory factors in uterine leiomyomas. J Assist Reprod Genet. 2019;36:2385‐2397. 31728810 10.1007/s10815-019-01582-y PMC6885490

[15] Yamagata Y , Nishino K , Takaki E , et al. Genome‐wide DNA methylation profiling in cultured eutopic and ectopic endometrial stromal cells. PLoS One. 2014;9:e83612. 24465385 10.1371/journal.pone.0083612 PMC3900404

[16] Maekawa R , Lee L , Okada M , et al. Changes in gene expression of histone modification enzymes in rat granulosa cells undergoing luteinization during ovulation. J Ovarian Res. 2016;9:15. 26979106 10.1186/s13048-016-0225-z PMC4793631

[17] Tamura I , Takagi H , Doi‐Tanaka Y , et al. Wilms tumor 1 regulates lipid accumulation in human endometrial stromal cells during decidualization. J Biol Chem. 2020;295:4673‐4683. 32098869 10.1074/jbc.RA120.012841 PMC7135999

[18] Kajimura T , Sato S , Murakami A , et al. Overexpression of carbonyl reductase 1 inhibits malignant behaviors and epithelial mesenchymal transition by suppressing TGF‐β signaling in uterine leiomyosarcoma cells. Oncol Lett. 2019;18:1503‐1512. 31423217 10.3892/ol.2019.10429 PMC6607169

[19] Nishimoto Y , Murakami A , Sato S , et al. Decreased carbonyl reductase 1 expression promotes tumor growth via epithelial mesenchymal transition in uterine cervical squamous cell carcinomas. Reprod Med Biol. 2018;17:173‐181. 29692675 10.1002/rmb2.12086 PMC5902461

[20] Suo G , Han J , Wang X , et al. Oct4 pseudogenes are transcribed in cancers. Biochem Biophys Res Commun. 2005;337:1047‐1051. 16229821 10.1016/j.bbrc.2005.09.157

[21] Bai M , Yuan M , Liao H , et al. OCT4 pseudogene 5 upregulates OCT4 expression to promote proliferation by competing with miR‐145 in endometrial carcinoma. Oncol Rep. 2015;33:1745‐1752. 25634023 10.3892/or.2015.3763

[22] Wang C , Qi S , Xie C , Li C , Wang P , Liu D . Upregulation of long non‐coding RNA XIST has anticancer effects on epithelial ovarian cancer cells through inverse downregulation of hsa‐miR‐214‐3p. J Gynecol Oncol. 2018;29:e99. 30207107 10.3802/jgo.2018.29.e99 PMC6189427

[23] Xu B , Gong X , Zi L , et al. Silencing of DLEU2 suppresses pancreatic cancer cell proliferation and invasion by upregulating microRNA‐455. Cancer Sci. 2019;110:1676‐1685. 30838724 10.1111/cas.13987 PMC6501038

[24] Wang L , Yu M , Zhao S . lncRNA MEG3 modified epithelial‐mesenchymal transition of ovarian cancer cells by sponging miR‐219a‐5p and regulating EGFR. J Cell Biochem. 2019;120:17709‐17722. 31161607 10.1002/jcb.29037

[25] Xiu YL , Sun KX , Chen X , et al. Upregulation of the lncRNA Meg3 induces autophagy to inhibit tumorigenesis and progression of epithelial ovarian carcinoma by regulating activity of ATG3. Oncotarget. 2017;8:31714‐31725. 28423647 10.18632/oncotarget.15955 PMC5458242

[26] Sheng X , Li J , Yang L , et al. Promoter hypermethylation influences the suppressive role of maternally expressed 3, a long non‐coding RNA, in the development of epithelial ovarian cancer. Oncol Rep. 2014;3:277‐285. 10.3892/or.2014.3208 24859196

[27] Domcke S , Sinha R , Levine DA , Sander C , Schultz N . Evaluating cell lines as tumour models by comparison of genomic profiles. Nat Commun. 2013;4:2126. 23839242 10.1038/ncomms3126 PMC3715866

[28] Wang A , Jin C , Li H , Qin Q , Li L . LncRNA ADAMTS9‐AS2 regulates ovarian cancer progression by targeting miR‐182‐5p/FOXF2 signaling pathway. Int J Biol Macromol. 2018;120:1705‐1713. 30268751 10.1016/j.ijbiomac.2018.09.179

